# Chiral Biomaterials for Nanomedicines: From Molecules to Supraparticles

**DOI:** 10.3390/pharmaceutics14091951

**Published:** 2022-09-15

**Authors:** Wookjin Jung, Junyoung Kwon, Wonjoon Cho, Jihyeon Yeom

**Affiliations:** 1 Department of Materials Science and Engineering, Korea Advanced Institute of Science and Technology (KAIST), Daejeon 34141, Korea; 2 Department of Biological Sciences, Korea Advanced Institute of Science and Technology (KAIST), Daejeon 34141, Korea; 3 Institute for Health Science and Technology, Korea Advanced Institute of Science and Technology (KAIST), Daejeon 34141, Korea; 4 Institute for NanoCentury, Korea Advanced Institute of Science and Technology (KAIST), Daejeon 34141, Korea

**Keywords:** chirality, nanomedicine, biomaterials, nanomaterials

## Abstract

Chirality, the property whereby an object or a system cannot be superimposed on its mirror image, prevails amongst nature over various scales. Especially in biology, numerous chiral building blocks and chiral-specific interactions are involved in many essential biological activities. Despite the prevalence of chirality in nature, it has been no longer than 70 years since the mechanisms of chiral-specific interactions drew scientific attention and began to be studied. Owing to the advent of chiral-sensitive equipment such as circular dichroism spectrometers or chiral liquid columns for chromatography, it has recently been possible to achieve a deeper understanding of the chiral-specific interactions and consequential impacts on the functionality and efficiency of nanomedicine. From this point of view, it is worthwhile to examine previously reported chiral biomaterials with their compositions and possible applications to achieve new paradigms of biomaterials. This review discusses chiral materials on various scales and their biological applications.

## 1. Introduction

Regarding the fact that the majority of organisms are composed of amino acids with left-handedness and DNA double helices with right-handedness, it is not surprising that most biological activities are very sensitive to the chirality of molecules that biosystems encounter [1,2]. For instance, there are several reports that the same molecule can taste and smell differently depending on its molecular chirality [3,4,5]. Considering that the distinctive taste or scent of molecules is determined based on several chemical reactions of our sensory receptors, this difference could be attributed to chiral-specific biochemical interactions of the receptors, which also consist of numerous chiral peptides [6]. Moreover, the chirality of a molecule can determine whether it will be therapeutic or toxic [7,8]. One of the most prominent cases is thalidomide, which was frequently prescribed for insomnia and morning sickness during the early 1960s [9]. When it was not known that the atomic configuration of (−)-(*S*)-thalidomide could interfere with vasculogenesis, the drug had been prescribed to numerous pregnant women, which led to serious birth defects [9,10]. Nevertheless, further studies based on stereochemistry revealed the enantioselective mechanism of vasculogenesis [10,11]. The detailed understanding of chiral-specific biochemical activities allowed this toxin to be reborn as an anticancer drug that inhibits the angiogenesis of malignant tumors. Besides thalidomide, several following studies have demonstrated that the pharmacological actions of various drugs are highly governed by their molecular chirality [12,13]. Therefore, discriminating a eutomer, a specific enantiomer of a chiral compound that is more potent, from the less potent distomer has been considered as a crucial step for designing novel drugs [14].

Meanwhile, nanomaterials have begun to emerge as a novel pharmaceutical platform, which had highly relied on therapeutic biomolecules [15]. Indeed, nanomedicine based on nanomaterials with tunable properties, including optical, magnetic and biological properties, could enable unprecedented therapeutic strategies with high drug efficiency and low side effects [16]. Nonetheless, several breakthroughs, such as enhanced cell uptake or selective targeting, have been required for nanomedicine to be clinically applicable [17,18]. One of the possible strategies to achieve these requirements is chiral-engineered nanomaterials. Similar to chiral biomolecules, several studies have suggested that interactions between biosystems and nanomaterials are heavily affected by the chirality of the nanomaterials, which could be utilized for improvements in nanomedicine. From this point of view, this review introduces recent advances in chiral biomaterials (Table 1) and perspectives.

## 2. Chiral Nanomaterials for Nanomedicine

**Figure 1:**
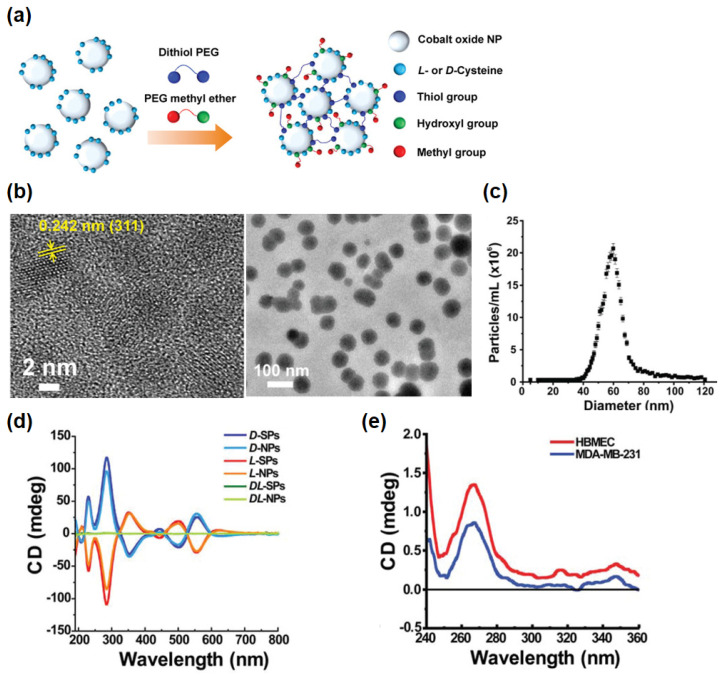
Characterization of chiral cobalt oxide NPs and SPs. (**a**) Schematic illustration of chiral NPs’ self-assembly into SPs. (**b**) Transmission electron microscopy (TEM) image of *L*-SPs and *D*-SPs. Yellow arrows indicate the (311) lattice fringe of chiral cobalt oxide NPs. (**c**) The size distribution curve of SPs. (**d**) CD spectra of SPs and NPs with different chirality. (**e**) CD spectra of lipids extracted from human cell (HBMEC and MDA-MB-231). Reprinted with permission from Ref. [19]. Copyright 2019 John Wiley and Sons.

Since the chirality of molecules governs their biological activities, as aforementioned, various studies using chiral nanomaterials for nanomedicine also have been conducted. Yeom et al. synthesized chiral supraparticles (SPs) for drug delivery systems based on previously reported chiral cobalt oxide (Co_3_O_4_) nanoparticles (NPs) [19]. In this study, the interactions between the surface chirality of the drug delivery system and the biological system were investigated and the overall performance of the chiral SPs was evaluated. The chiral cobalt SPs were prepared using *L-* and *D-*cysteine as chiral agents and *DL-*cysteine was used for their control, achiral cobalt SPs (Figure 1a). The prepared SPs had a size of around 60 nm (Figure 1b,c) and their optical activity was confirmed via circular dichroism (CD) spectroscopy (Figure 1d). The authors hypothesized that SPs with *D-*cysteine (*D-*SPs) would interact with the cellular membrane more effectively than SPs with *L-*cysteine (*L-*SPs) since both *D-*SPs and lipids from the cell membrane showed a positive CD signal at around 270 nm, while *L-*SPs showed a negative CD signal at the same wavelength (Figure 1e).

**Figure 2:**
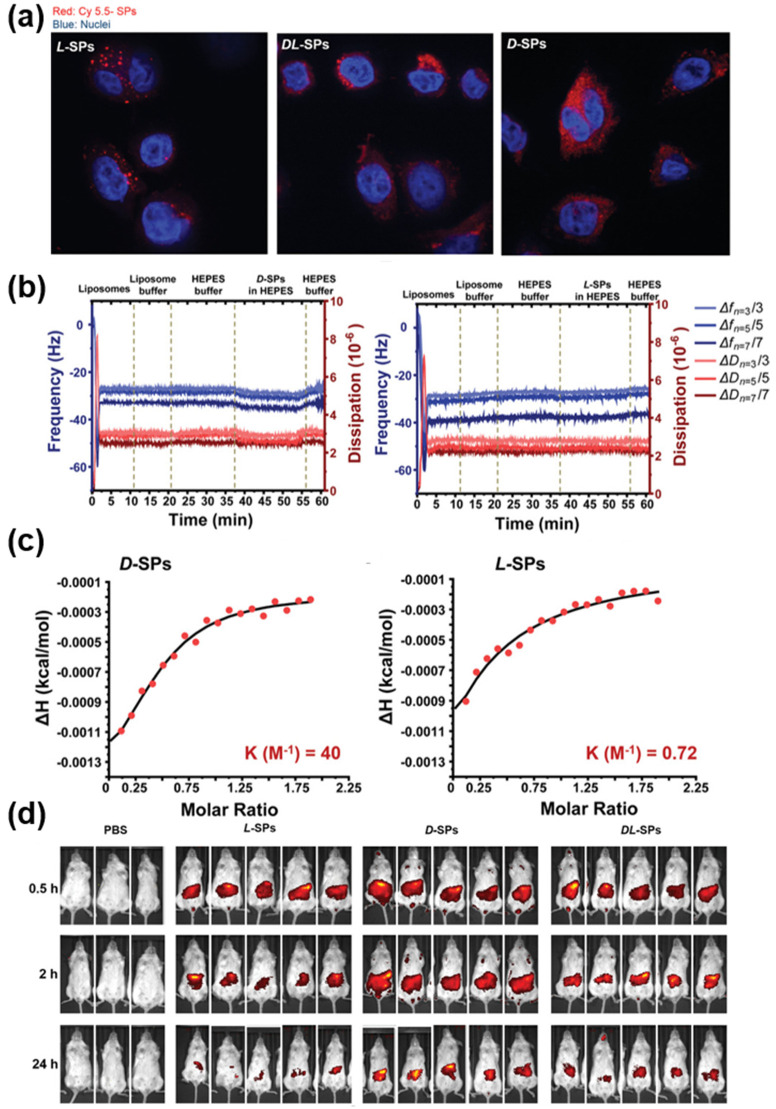
(**a**) Confocal images of HeLa cell nuclei (blue) and internalized *D-*, *L-* and *DL-*SPs (red). (**b**) QCM-D monitoring of *D-* and *L-*SPs’ adhesion on lipid bilayers. (**c**) ITC experiments for *D-* and *L-*SPs in liposome dispersions. (**d**) In vivo imaging system images of mice after intravenous injection of phosphate-buffered saline, *L-*, *D-* and *DL-*SPs. Reprinted with permission from Ref. [19]. Copyright 2019 John Wiley and Sons.

To confirm the hypothesis, the authors exposed HeLa cells to SPs with fluorescent dye for 24 h before confocal imaging was carried out. The results revealed that SPs with the same handedness (*D-*SPs) as the cell membranes exhibited better adhesion to phospholipid cellular membranes, which led to better cellular internalization (Figure 2a). The stronger adhesion of *D-*SPs to liposomes, which were analogous to the cellular membrane, also was examined by performing quartz crystal microbalance with dissipation (QCM-D) measurements and isothermal titration calorimetry (ITC) measurements (Figure 2b,c). These results confirmed that *D-*SPs were more adhesive to phospholipid membranes and this chiral-specific preference could be attributed to the difference in thermodynamic binding affinity between SPs and lipid molecules. Subsequently, the effect of chirality was tested through an in vivo study. SPs conjugated with fluorescent dye were injected intravenously into mice. After 0.5 h, 2 h and 24 h, the distribution of injected SPs was measured by an in vivo imaging system (Figure 2d). The result showed that SPs with *D-*handedness exhibited a longer biological half-life compared to their counterpart with *L-*handedness. The authors suggested that the *D-*amino acids on the SPs could act as a stealth sheath layer, which inhibited enzymes in blood plasma from digesting the SPs. Despite the innate toxicity of cobalt-based nanomedicine, this work could be considered as a pioneering study demonstrating that chiral surface engineering can be applied for the design of high-efficiency drug delivery systems [32].

**Figure 3:**
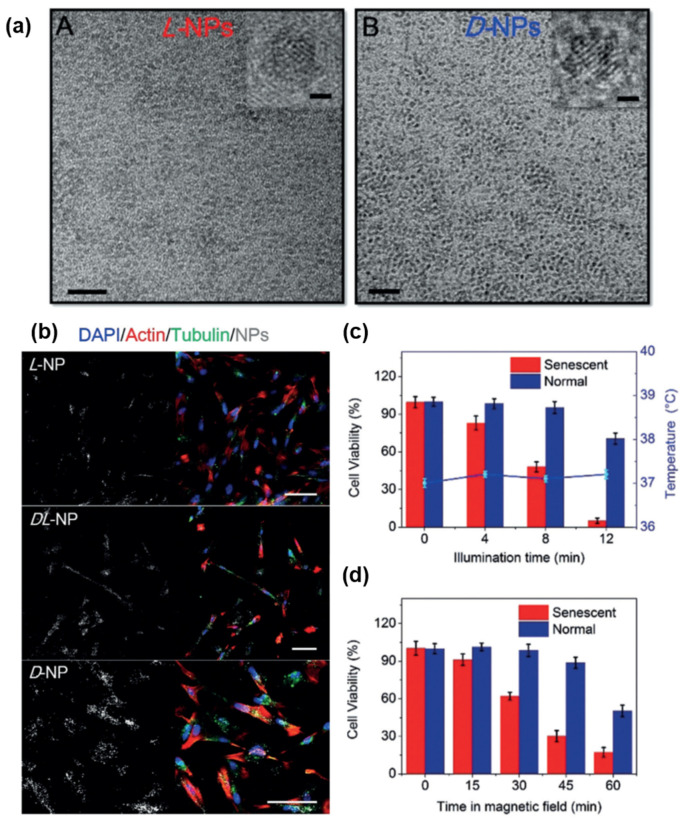
(**a**) TEM images of (A) *L-* and (B) *D-*penicillamine stabilized Cu_x_Co_y_S NPs, scale bar = 20 nm. The upper-right images are high-resolution images of corresponding NPs, scale bar = 1 nm. (**b**) Confocal images of *L-*, *DL-* and *D-*NPs in senescent cells, scale bar = 100 μm. (**c**) Cell viability of *D-*NP internalized senescent cells with different NIR illumination times. (**d**) Cell viability of *D-*NP internalized senescent cells after being treated with AMF at different time points. Reprinted with permission from Ref. [20]. Copyright 2020 John Wiley and Sons.

Similarly, Li et al. synthesized chiral copper cobalt sulfide (Cu_x_Co_y_S) nanoparticles for the selective elimination of senescent cells [20]. The NPs were prepared by using *L-*, *DL-* and *D-*penicillamine as surface chiral ligands (Figure 3a). In detail, induced senescent cells were incubated with the NPs and then confocal imaging was carried out. The result showed that senescent cells accepted NPs covered with *D-*penicillamine (*D-*NPs) more efficiently than NPs covered with *L-*penicillamine (*L-*NPs) (Figure 3b). Aside from the chiral effect, it was reported that intracellular Cu_x_Co_y_S NPs released reactive oxygen species (ROS), which triggered the apoptosis of senescent cells under near-infrared (NIR) radiation. It was also reported that the mechanical movement of the NPs triggered the apoptosis of the cells under an alternating magnetic field (AMF) since the NPs exhibited moderate ferromagnetism. Considering the apoptotic effect of the NPs, the authors claimed that *D-*NPs could selectively and effectively induce the apoptosis of senescent cells under NIR radiation and AMF (Figure 3c,d).

**Figure 4:**
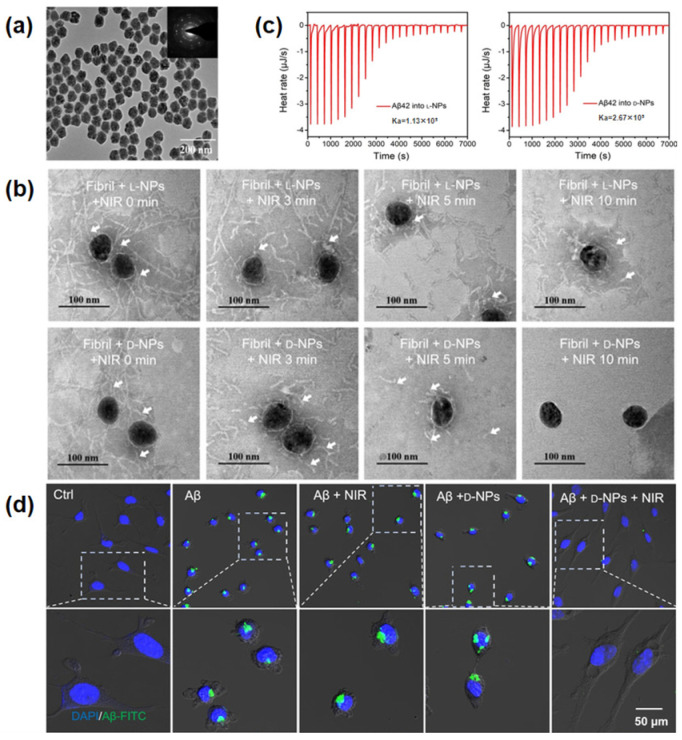
(**a**) TEM image and selected area electron diffraction pattern of chiral Fe_x_Cu_y_Se NPs. (**b**) TEM images of preformed Aβ42 fibrils at different times (0, 3, 5 and 10 min) of treatment with *L-*/*D-*NPs and NIR irradiation. Arrows indicate Aβ42 fibrils (**c**) ITC experiments for *L-* and *D-*NPs in Aβ42 protein solution. (**d**) Confocal images showing Aβ42 (green) adhesion onto the membranes of MN9D cells treated with NIR, *D-*NPs, as well as *D-*NPs and NIR. Nuclei were stained with DAPI (blue). Reprinted with permission from Ref. [26]. Copyright 2020 John Wiley and Sons.

Chiral nanoparticles can also be applied for regulating the formation of peptide fibrils, which are much smaller than cellular scale [22,26]. Zhang et al. synthesized chiral iron copper selenide (Fe_x_Cu_y_Se) NPs decorated with *L-* and *D-* penicillamine (Figure 4a) [26]. Since Fe_x_Cu_y_Se NPs were also known to generate reactive oxygen species under NIR radiation, they suggested that the chiral NPs and reactive oxygen species from them would prohibit the formation of Aβ42 fibrils, a well-known biomarker of Alzheimer’s disease (AD). To verify the hypothesis, mixtures of Fe_x_Cu_y_Se NPs conjugated with *L-*/*D-* penicillamine (*L-*/*D-*NPs) and Aβ42 fibrils were irradiated with NIR light for 10 min. Transmission electron microscopy (TEM) images exhibited that the fibrils with *D-*NPs were disintegrated after NIR radiation, while the fibrils with *L-*NPs remained (Figure 4b). To elucidate this chiral-specific disintegration, ITC experiments were conducted. ITC results indicated that the binding affinity between Aβ42 fibrils and *D-*NPs was two times higher than the binding affinity between the fibrils and *L-*NPs (Figure 4c). Moreover, the authors showed that injecting the *D-*NPs into the brains of AD mouse models could reduce the concentration of Aβ42 fibrils and alleviate their neurotoxicity (Figure 4d).

**Figure 5:**
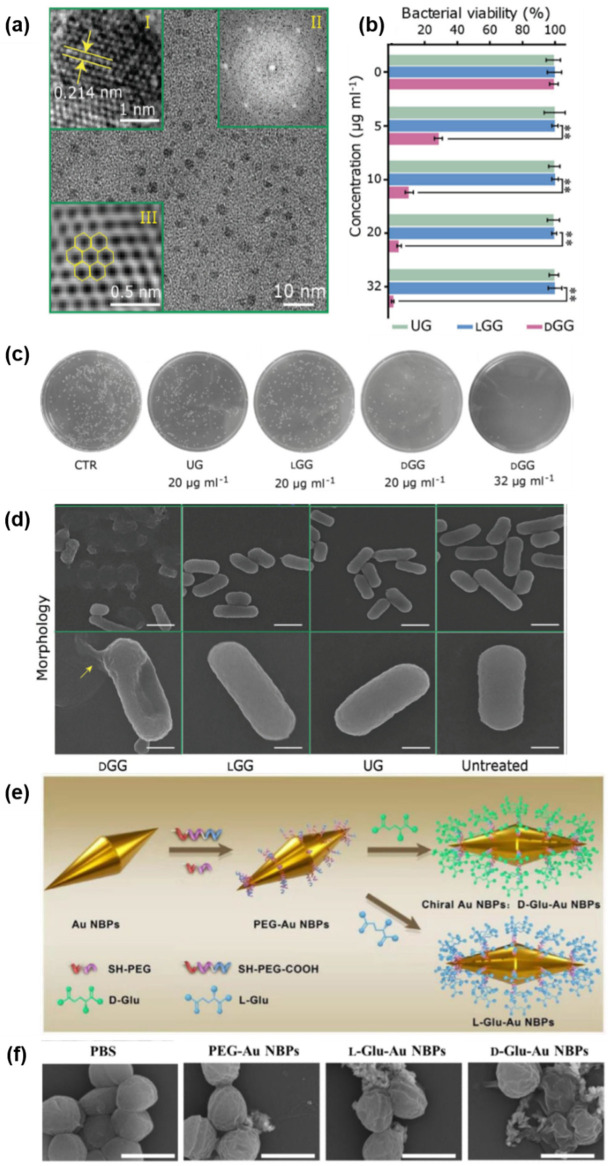
(**a**) TEM images of *D-*GGs. Inset I, II and III are the high-resolution TEM, corresponding fast Fourier transform (FFT) and reverse FFT images of *D-*GGs, respectively. Yellow arrows in inset I indicate the (100) lattice fringe of chiral graphene QDs and yellow hexagons in inset III indicate hexagonal crystalline structure of the QDs. (**b**) Antimicrobial activities of chiral GQDs and UGs against *S. aureus* evaluated by a standard plate count method, ** *p* < 0.01. (**c**) Antimicrobial activities of chiral GQDs and UGs against *E. coli*. (**d**) SEM images of *E. coli* after incubating *D-*GGs, *L-*GGs, UGs and normal saline as the control for 3 h, respectively, scale bar = 1 μm (first low), 500 nm (second row, yellow arrow indicates the leaked cellular content). (**e**) Schematic illustration of *D-*/*L-*Glu-Au NBP fabrication. (**f**) SEM images of *S. epidermidis* treated by phosphate-buffered saline (PBS), PEG-Au NBPs and *L-*/*D-*Glu-Au NBPs for 3 h, scale bar = 1 μm. Reprinted with permission from Refs. [21,27]. Copyright 2016 John Wiley and Sons. Copyright 2020 Elsevier.

Xin et al. focused on *D-*glutamic acid, which is an essential biomolecule for bacteria to synthesize peptidoglycan [21]. The authors synthesized graphene quantum dots (GQDs) by pyrolysis of citric acid with the *L-* and *D-* glutamic acid, which functionalized the GQDs (Figure 5a). They hypothesized that GQDs covered with *D-*glutamic acid (*D-*GGs) would inhibit the activity of MurD ligase, a crucial enzyme for peptidoglycan synthesis, while GQDs covered with *L-*glutamic acid (*L-*GGs) would not. To confirm the hypothesis, *Escherichia coli* (*E. coli*, Gram-negative) and *Staphylococcus aureus* (*S. aureus,* Gram-positive) were incubated with *L-*GGs, *D-*GGs and unfunctionalized GQDs (UGs) for a control experiment. The result revealed that *D-*GGs displayed dose-dependent antibacterial activity toward both *E. coli* and *S. aureus* (Figure 5b,c). Then, scanning electron microscopy (SEM) showed that *D-*GGs inhibited the formation of cell walls, which were composed of peptidoglycan, while *L-*GGs and UGs did not (Figure 5d). The damage to cell walls contributed to the leakage of the cellular content of *D-*GG-treated bacteria, which led to their death.

A similar study using gold nanoparticles functionalized with chiral glutamic acid was also conducted by Zhang et al. [27]. They synthesized gold nanobipyramids (Au NBPs) stabilized by thiolated polyethylene glycol (PEG) with carboxyl group terminals. Then, *L-* and *D-*glutamic acids were conjugated with terminal carboxyl groups (Figure 5e). The synthesized *L-* and *D-* glutamic acid-conjugated gold nanobipyramids (*L-*/*D-*Glu-Au NBPs) were added to bacterial suspensions of *Staphylococcus epidermidis* (*S. epidermidis*) to evaluate the antibacterial properties of the chiral NBPs. Similar to the case of chiral graphene quantum dots, SEM images showed that the cell walls of *S. epidermidis* incubated with *D-*Glu-Au NBPs were damaged, while those of *L-*Glu-Au NBP-treated *S. epidermidis* were relatively intact (Figure 5f). Since the antibacterial mechanisms of these chiral nanomedicines are significantly different from the antibacterial mechanisms of conventional β-lactam antibiotics, chiral nanoantibiotics would be a novel approach for treating antibiotic-resistant bacteria.

**Figure 6:**
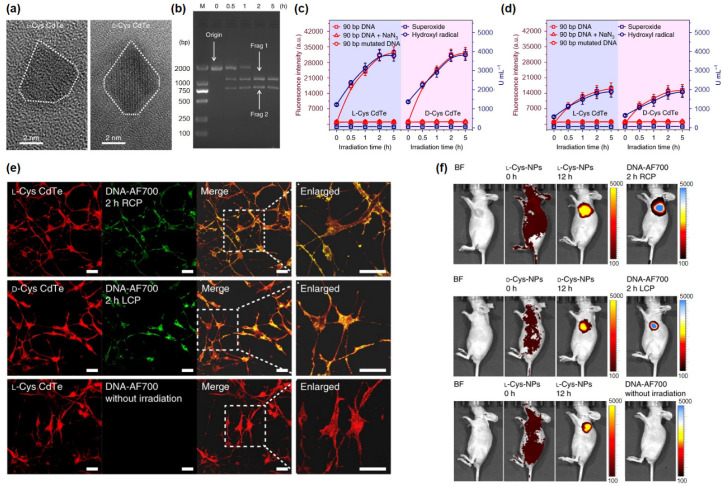
(**a**) TEM images of *L-*/*D-*Cys CdTe NPs. (**b**) Electrophoresis images of *L-*Cys CdTe NPs with 1839 bp DNA illuminated RCP for 2 h. The two DNA fragments after cleavage are denoted by frag 1 and frag 2. (**c**) ROS production with 90 bp DNA, mutated 90 bp DNA and 90 bp DNA with ROS inhibitor (NaN_3_) in *L-*Cys CdTe NPs under RCP and *D-*Cys CdTe NPs under LCP. (**d**) ROS production in *L-*Cys CdTe NPs under LCP and *D-*Cys CdTe NPs under RCP. (**e**) Confocal images of neural stem cells incubated with *L-*/*D-*Cys CdTe NPs under RCP/LCP and without irradiation, scale bar = 20 μm. (**f**) In vivo images of nude mice after *L-*/*D-*Cys CdTe NPs injected with irradiation under RCP/LCP for 2 h and without irradiation. Reprinted with permission from Ref. [23]. Copyright 2018 Springer Nature.

While the aforementioned studies focused on a chiral selective interaction between nanomaterials and the biosystem itself, there have been several attempts to utilize the optical activity of chiral materials for nanomedicine [23,24]. Sun et al. reported that chiral cysteine-modified CdTe nanoparticles could be utilized for cleaving specific sequences of DNA double strands (Figure 6a) [23]. In detail, the authors demonstrated that chiral CdTe NPs produced ROS, which cleaved phosphodiester bonds within DNA backbones by oxidation, under 405 nm light radiation (Figure 6b). When circularly polarized light (CPL) was illuminated, the amount of produced ROS was affected by the handedness of the illuminated CPL because the chiral NPs were optically active. For instance, the number of hydroxyl radicals produced by *L-*cysteine-modified CdTe NPs (*L-*Cys-CdTe) under right-handed circularly polarized light (RCP) illumination was doubled, compared to that under left-handed circularly polarized light (LCP) illumination (Figure 6c,d). These results suggested that the activities of abiotic nanozymes based on chiral nanomaterials could be controlled by CPL radiation. Indeed, the authors successfully demonstrated the CPL-−induced DNA cleavage in living cells and in vivo, which were confirmed by fluorescent imaging (Figure 6e,f). Since DNA itself and its transcription could be easily affected by a considerable number of small molecules, it has been difficult to find appropriate molecular drugs for precisely targeting specific DNA sequences [33,34]. Considering the fact that medication based on chiral NPs seems to be an emerging alternative, although the reported photocleavage effect of chiral CdTe NPs was limited to a specific recognition site (GATATC), this study suggested a new design strategy for highly stereospecific and controllable abiotic nanoenzymes, which would be crucial for gene therapy [35].

**Figure 7:**
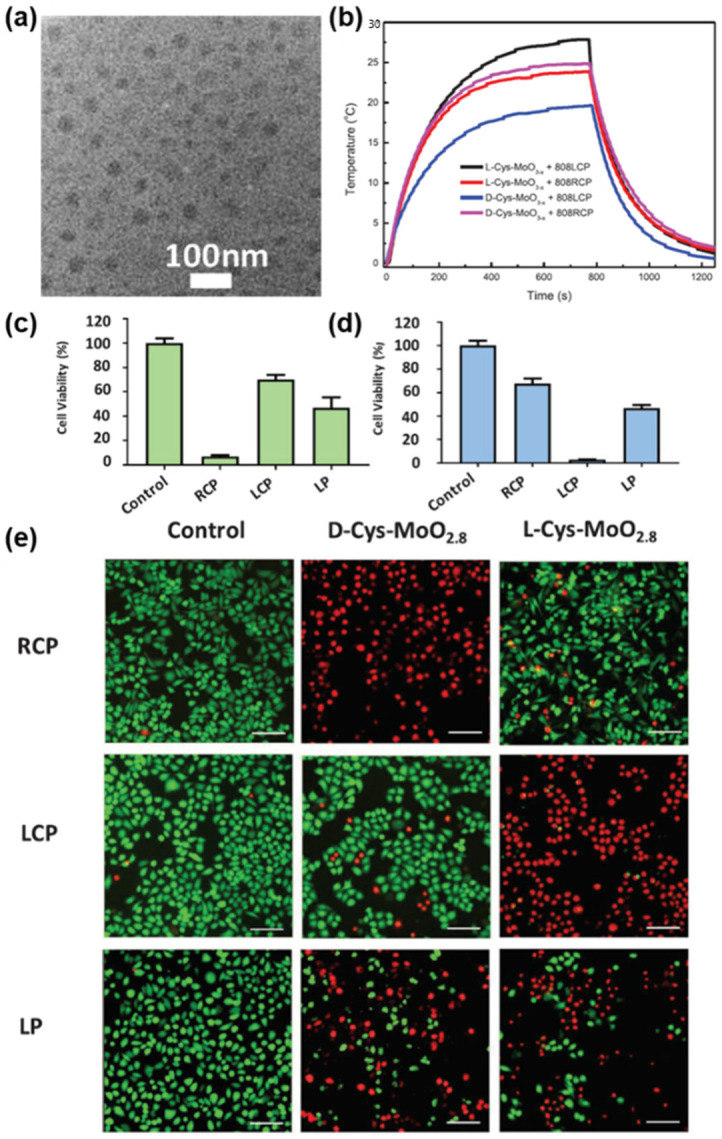
(**a**) TEM image of *L-*Cys-MoO_3−x_ NPs. (**b**) Temperature versus time curves of *L-*/D-Cys-MoO_3−x_ NPs under 808 nm LCP/RCP radiation. (**c**) The viability of HeLa cells incubated with D-Cys-MoO_3−x_ NPs and (**d**) *L-*Cys-MoO_3−x_ NPs. (**e**) Fluorescence microscopy images of live (green) and dead (red) HeLa cells after RCP, LCP and LP light irradiation, scale bar = 100 μm. Reprinted with permission from Ref. [24]. Copyright 2019 John Wiley and Sons.

Li et al. utilized the optical properties of chiral molybdenum oxide (MoO_3−x_) NPs in photothermal therapy (PTT) for cancer treatment [24]. The chiral NPs were obtained by the substoichiometric reduction of cysteine molecules, which also acted as a chiral capping agent (Figure 7a). Similar to the studies conducted by Sun and coworkers, the amount of heat generated by the chiral MoO_3−x_ NPs varied with the combination of the handedness of the NPs and CPL (Figure 7b) [23]. An in vitro study using HeLa cells for chiral PTT followed. A standard Cell Counting Kit-8 (CCK-8) assay revealed that 93.01% of HeLa cells incubated with *D*-cysteine-capped chiral MoO_3−x_ NPs (*D*-Cys-MoO_3−x_ NPs) were dead after 15 min of 808 nm RCP irradiation, while only 29.98% of the cells were dead under LCP irradiation (Figure 7c). This chiral-specific cell viability tendency was inverted when HeLa cells were incubated with *L-*cysteine-capped chiral MoO_3−x_ NPs (*L-*Cys-MoO_3−x_ NPs); 32.25% of the cells were dead under RCP irradiation, while the fatality rate reached 96.95% when LCP was irradiated (Figure 7d). These chiral-specific PTT results were also confirmed by confocal microscopy (Figure 7e).

## 3. Chiral Biomaterials with Supramolecular Structures

Unlike nanoparticles whose atomic components are held together by primary bonds, materials with supramolecular structures consist of monomers organized by noncovalent, intermolecular binding interactions [36]. Supramolecular materials have recently drawn attention since they have emergent properties that their unorganized subunits do not possess [37]. Indeed, this supramolecular emergence could be easily found in nature. One example is collagen, the main protein component of various types of connective tissue. A single collagen microfibril has right-handed chirality, while its subunit polypeptide strands have left-handed chirality [38]. Considering that this emergent supramolecular right-handed chirality of collagen fibrils plays a pivotal role in cell adhesion, biomaterials with supramolecular chirality are worthwhile to investigate further [39].

**Figure 8:**
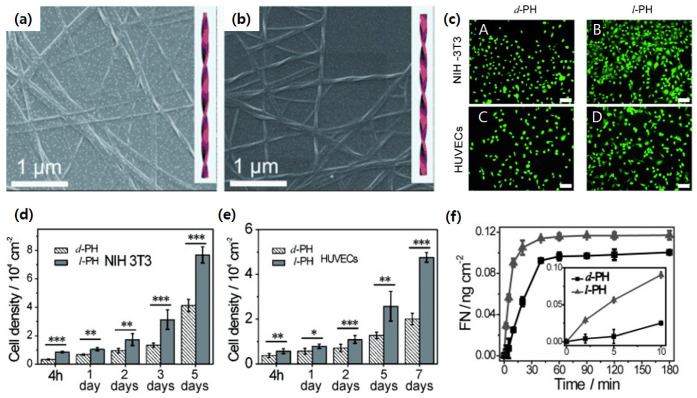
SEM images and reconstructed model of (**a**) right-handed helical fibers (*d-*PH) and (**b**) left-handed helical fibers (*l-*PH). (**c**) Fluorescence microscopy images of NIH/3T3 cells after culture for 3 days (A,B) and HUVECs after culture for 5 days (C,D) on *d-/l-*PH, scale bar = 100 μm. Cell density data for (**d**) NIH/3T3 cells and (**e**) HUVECs on *d-/l-*PH films after incubation. *, **, and *** data show significant differences (ANOVA: * *p* ≤ 0.05, ** *p* ≤ 0.005, *** *p* ≤ 0.001). (**f**) Time-dependent adsorption of fibronectin (FN) on *d-/l-*PH films. Inset: Adsorption of FN on *d-/l-*PH films from 0 to 10 min. Reprinted with permission from Ref. [30]. Copyright 2014 John Wiley and Sons.

One of the applications in the field of supramolecular chiral biomaterials is synthetic hydrogel networks for the 3D extracellular matrix (ECM) [30,31,40]. Liu et al. hypothesized that cell adhesion and proliferation would be determined by the chirality of the polymeric matrix where cells grew [30]. They synthesized chiral nanofibers based on chiral 1,4-benzenedicarboxamide phenylalanine derivative (PH) monomers. When the PH monomers were mixed with an aqueous medium for cells, a hydrogel with helical fibers was spontaneously formed due to hydrophobic benzene ring moieties (Figure 8a,b) [41]. Mouse fibroblast cells (NIH/3T3) and human endothelial cells (HUVECs) were then cultured onto the chiral hydrogels. It was found that cells cultured in the left-handed hydrogel showed higher adhesion and density, regardless of the cell line (Figure 8c–e). Further studies using fibronectin, a protein promoting cell adhesion, revealed that a larger amount of fibronectin was adsorbed by left-handed fibers than right-handed fibers (Figure 8f). Considering that adsorbed fibronectin acted as a cellular anchor, the left-handed helices could provide more cellular anchoring sites compared to right-handed helices [42].

**Figure 9:**
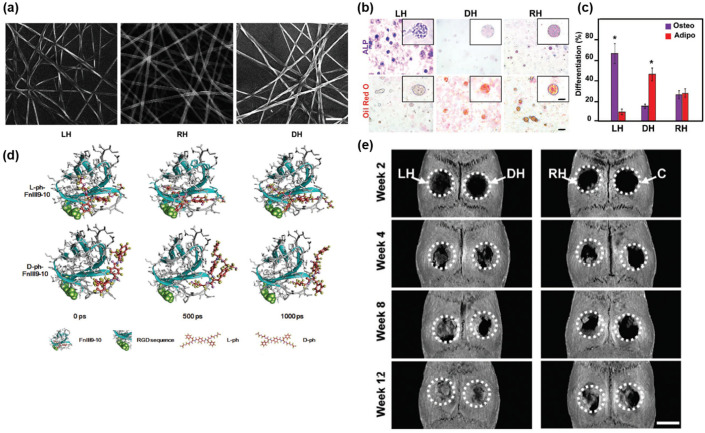
(**a**) SEM images of left-handed matrix (LH), racemic matrix (RH) and right-handed matrix (DH), scale bar = 200 nm. (**b**) Alkaline phosphatase (ALP) and lipid droplet (Oil Red O) staining results. (**c**) Differentiation percentage of mesenchymal stem cells (MSCs). * *p* < 0.05, *t*-test. (**d**) Snapshots of the molecular dynamic simulations illustrating the effect of chirality on fibronectin (Fn) tethering. (**e**) CT images of bone regeneration in rat cranial defects after MSC/matrix implantation, scale bar = 5 mm. Reprinted with permission from Ref. [31]. Copyright 2019 John Wiley and Sons.

The stereospecific interaction between chiral suprastructures and fibronectin can also dictate cell differentiation. Wei et al. demonstrated that the cell differentiation of mesenchymal stem cells (MSCs) was governed by the handedness of the surrounding matrix [31]. Similar to Liu and colleagues, the authors fabricated chiral matrixes by using 4-benzenedicarboxamide phenylalanine derivatives as monomers for cell culture (Figure 9a). After 14 days of incubation, the cell phenotype of cultured MSCs was analyzed by fluorescent staining. It revealed that MSCs cultured in the left-handed matrix (LH) produced more alkaline phosphatase, which indicated the osteogenesis of MSCs, than the cells cultured in the right-handed counterpart. Meanwhile, MSCs cultured in the right-handed matrix (DH) produced more lipids, which were significant clues regarding the adipogenesis of stem cells (Figure 9b,c). The mechanism of the chiral-specific differentiation was attributed to stereospecific interactions between fibronectin and the chiral monomers, according to molecular dynamic (MD) simulations (Figure 9d). The MD simulation results demonstrated that fibronectin had a higher binding affinity for left-handed monomers than right-handed monomers, which caused more fibronectin to be absorbed on LH. Considering that fibronectin was recognized by a cellular mechanosensory protein (Itgα5), the upregulated Itgα5 triggered the osteogenesis of MSCs in LH [43]. Furthermore, the authors demonstrated that chiral-specific osteogenesis occurred in vivo. MSCs in chiral (LH/DH) and achiral (racemic matrix, RH) matrices were injected into defected rat cranial bones. After 12 weeks, MSC/LH−injected rats were fully recovered, while other groups of rats were not fully recovered (Figure 9e). These studies using chiral hydrogels clearly demonstrated that biological interactions, such as wound healing, are deeply governed by nanoscale 3D structures including chirality, which have not been fully investigated.

Similar to chiral biomaterials with nanoscale, there have been several attempts to utilize the optical activity of chiral supramolecular materials for biomedical purposes recently [25,28,29]. Indeed, optical activity in the NIR region is one of the most promising properties of supramolecular chiral materials for biomedical applications. While either ultraviolet (UV) light or visible (vis) light has a short attenuation length, which is a critical drawback for biomedical applications, the longer attenuation length of NIR light enables photoresponsive biomaterials to be used from diagnostics to treatment [44,45,46].

**Figure 10:**
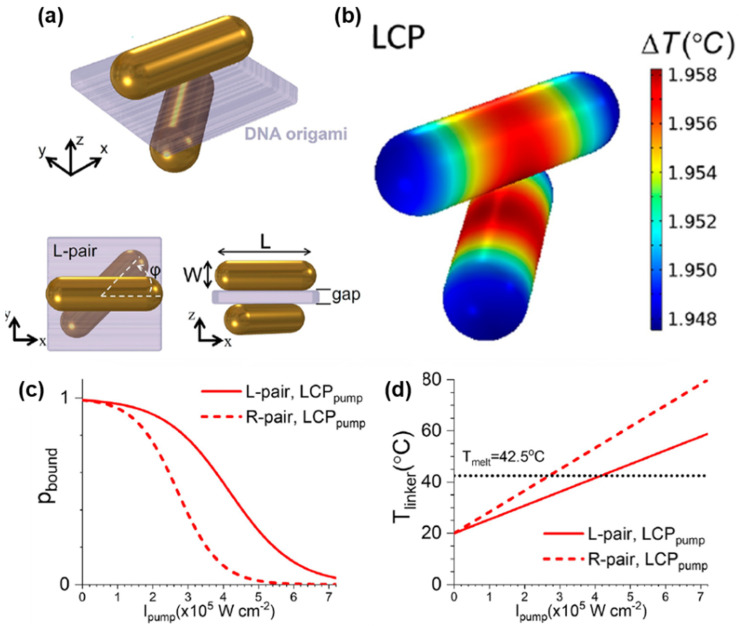
(**a**) Schematics of a pair of left-handed gold nanorods separated by DNA origami. (**b**) A surface temperature map of calculated temperature increases under 790 nm LCP irradiation. (**c**) Probability distributions of finding left-handed dimers (L-pair) and right-handed dimers (R-pair) under 790 nm LCP irradiation. (**d**) The local temperature at the point between nanorod dimers under 790 nm LCP irradiation. Reprinted with permission from Ref. [25]. Copyright 2021 American Chemical Society.

For instance, Ávalos-Ovando et al. focused on chiral-specific photothermal heating induced by a pair of gold nanorods (NRs) formed with a sheet of DNA origami under CPL irradiation (Figure 10a) [25]. Unlike nanoparticles without chirality, the photothermal activity induced by CPL of these chiral gold structures was highly localized within the gap between a pair of NRs, so-called a photothermal hotspot (Figure 10b) [47,48]. When CPL was irradiated, heat generated within the hotspots caused the chiral structures to break, since the DNA origami layers between nanorods were disintegrated by the heat. It was demonstrated that when 790 nm LCP was irradiated, a pair of Au NRs with a right-handed structure (R-pair) were easier to break into two achiral Au NRs, compared to the left-handed pair (L-pair) (Figure 10c,d). This result was attributed to the fact that 790 nm LCP was more efficiently absorbed by the R-pair than the L-pair, which was confirmed by circular dichroism spectroscopy. Considering that the controlled release of drugs can be achieved by using thermoresponsive polymers [49], the CPL-sensitive photoheating could be applied to drug delivery systems.

**Figure 11:**
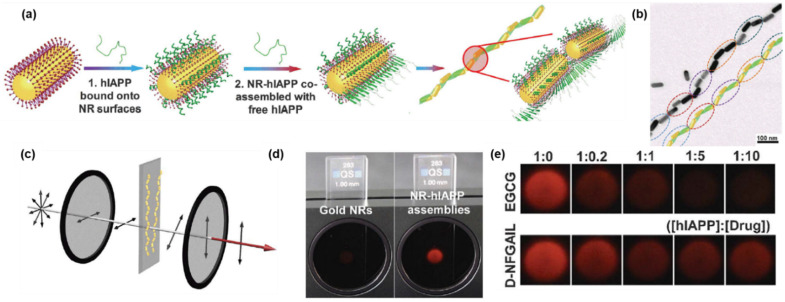
(**a**) Schematics of the assembly process of hIAPP monomers with NRs. (**b**) TEM image and reconstructed model of NR-hIAPP assemblies. (**c**) Schematics for cross-polarization optical cell. (**d**) Photographs of pure Au NRs and NR-hIAPP assemblies under cross-polarization conditions. (**e**) Photographs of the NR-hIAPP coincubated with two model drugs in different concentrations under cross-polarization conditions. Reprinted with permission from Ref. [29]. Copyright 2021 the American Association for the Advancement of Science.

Lu et al. suggested a novel drug discovery protocol using chiral Au NR assemblies decorated along human islet amyloid polypeptide (hIAPP) fibrils [29]. They demonstrated that when premade hIAPP-bounded Au NRs were mixed with free hIAPP, left-handed helical assemblies of gold nanorods were self-assembled (Figure 11a,b). When linearly polarized light was propagated through the assembled chiral Au NRs, the electric field vector of propagating light would be rotated since the assembled chiral NRs had optical activity at the NIR region [50]. As the Au NRs were assembled in a highly ordered chiral manner, the intensity of the rotating light increased, which could be measured by a cross-polarization optical cell (Figure 11c,d). The authors suggested that potential drugs for inhibiting amyloid-fiber synthesis could be screened by using the chiral Au NR assemblies. For instance, the authors tested two molecules, epigallocatechin gallate (EGCG) and hIAPP-derived peptide (*D-*NFGAIL), for amyloid-fiber inhibitors (Figure 11e). When EGCG was mixed with hIAPP-bound Au NRs and free hIAPPs, the self-assembly of chiral Au NRs interfered, which was indicated by the weak intensity of transmitted light [51]. Meanwhile, when *D-*NFGAIL was mixed with the hIAPP-Au NRs and free hIAPPs, the self-assembly was less effectively inhibited. The authors claimed that this protocol could be more effective compared to traditional fluorescence-based drug screening methods since auto-fluorescent signals from various biomolecules interfere with the signals from target drugs.

**Figure 12:**
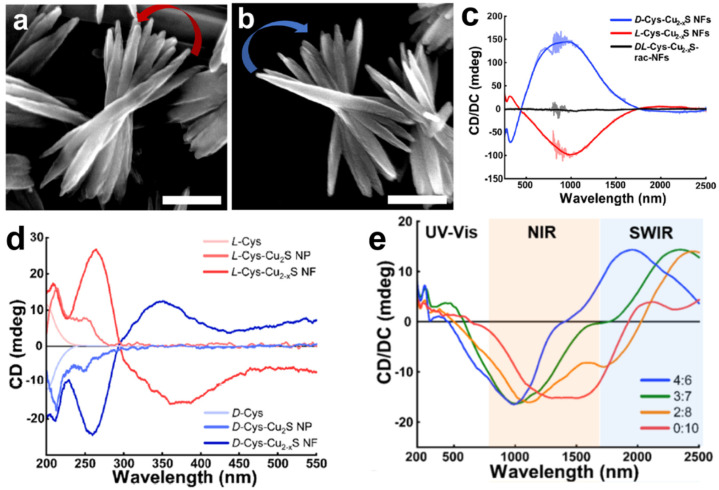
SEM images of (**a**) *L-*Cys-Cu_2−x_S NFs and (**b**) *D-*Cys-Cu_2−x_S NFs, scale bar = 500 nm. (**c**) CD spectra of thin films of NFs on a quartz wafer. (**d**) CD spectra demonstrating chirality transfer from *L-*/*D-*cysteine to the corresponding NFs. (**e**) CD spectra of chiral NFs with different chiral ligand molar ratios (thioglycolic acid: *L-*cysteine). Reprinted with permission from Ref. [28]. Copyright 2021 American Chemical Society.

Park et al. reported chiral copper sulfide (Cu_2−x_S) supramolecular nanoflowers (NFs) with broad optical activity in the UV–vis–NIR–short-wave infrared (SWIR) region using cysteine as chiral ligands (Figure 12a–c) [28]. The IR region has been considered important for biological applications since it is a so-called biologically transparent region where bio-species such as muscle, bone, fat, etc., hardly absorb light [52]. It was revealed that the molecular chirality of a single cysteine molecule was transferred to the nanoscale chirality of NPs and SPs, and the nanoscale chirality ultimately determined the microscale chirality of NFs through self-assembly processes (Figure 12d). Moreover, the authors emphasized that this chirality transfer process could be fully controlled by varying the growth times and the molecular ratio of the chiral initiator (Figure 12e). The controllability of chirality over a broad range of spectra would be regarded as a crucial technology for bioimaging, including multi-channel imaging or time-series imaging.

## 4. Conclusions and Outlook

Unlike materials for other technologies, strict constraints are imposed for the design of novel biomaterials for nanomedicine [53]. Base materials for nanomedicine have been highly limited; thus, finding a niche for designing biomaterials with dramatically enhanced functionalities has been regarded as a time-consuming and high-risk task. However, several remarkable studies have shown that a small tweak in the chiral conformation of materials could lead to considerable improvements in properties that are crucial for biomaterials, providing more degrees of freedom [19,21,27,30]. Meanwhile, other studies demonstrated novel approaches for controlling the pharmacological actions of biomaterials by using their chiral-specific interactions [23,24,25,29,31].

Discovering and designing new drugs relies heavily on the biochemical properties of the small molecules [54]. Though nanomedicines based on nanomaterials with various physicochemical features seem intriguing, critical drawbacks include low therapeutic efficacy or high cytotoxicity [55,56]. In this stalemate, the concept of chiral engineering would offer a brand-new breakthrough for designing high-functioning biomaterials such as nano-scaffolds with enhanced cell adhesion for wound healing or targeted drug delivery systems with improved cellular internalization while minimizing side effects [19,57].

Nonetheless, there have been several limitations for reported chiral-engineered biomaterials. Above all, a general and consensual explanation of how the biosystem perceives and reacts to surrounding chiral environments has not been fully suggested yet. Though some studies have tried to examine and unravel these chiral-specific interactions based on thermodynamic simulations, the results are somewhat incoherent and highly limited to molecular scales [2,29,31]. If comprehensive principles for chiral-specific interactions between nanomaterials and biosystems are clearly demonstrated, the design of novel chiral biomaterials with various functionalities would be possible. Based on the design principle of chiral biomaterials, multi-functional nanomedicine would be supplied on demand, which would ultimately lead to the realization of precision medicine.

## Figures and Tables

**Table 1:** Chiral biomaterials with various scales and their possible applications.

Base Materials	Chiral Agents	Size (nm)	Possible Applications	Ref.
Co_3_O_4_ nanoparticles (NPs)	*L-*/*D-*cysteine	2–3	Drug delivery system	[2,19]
Co_x_Cu_y_S NPs	*L-*/*D-*penicillamine	2–3	Selective senescent cell elimination	[20]
Graphene quantum dots	*L-*/*D-*glutamic acid	3	Anti-microbial activity	[21]
Carbon dots	*L-*/*D-*lysine	4	Reducing toxicity of β-amyloid fibril	[22]
CdTe NPs	*L-*/*D-*cysteine	4–5	Site-selective DNA photocleavage	[23]
MoO_3−x_ NPs	*L-*/*D-*cysteine	21–22	Photothermal therapy	[24]
Au nanorod dimers	DNA origami	40	Controlling drug release	[25]
Fe_x_Cu_y_Se NPs	*L-*/*D-*penicillamine	40–50	β-amyloid fibril elimination	[26]
Au bipyramid NPs	*L-*/*D-*glutamic acid	110 (length) 35 (width)	Anti-microbial activity	[27]
Cu_2−x_S nanoflowers	*L-*/*D-*cysteine	1500–2000	Multi-channel bioimaging	[28]
Au nanorod assemblies	Human islet amyloid polypeptides	Several μm (length) 50 (width)	Drug screening	[29]
1,4-benzenedicarboxamide phenylalanine hydrogel	*L-*/*D-*1,4-benzenedicarboxamide phenylalanine derivative	Several μm (length) 50–60 (width)	Scaffolds for wound healing	[30,31]
